# Analysis of Osteoblast Differentiation on Polymer Thin Films Embedded with Carbon Nanotubes

**DOI:** 10.1371/journal.pone.0129856

**Published:** 2015-06-15

**Authors:** Jin Woo Lee, Jin-Woo Park, Dongwoo Khang

**Affiliations:** 1 Department of Molecular Medicine, School of Medicine, Gachon University, Incheon, South Korea; 2 Department of Periodontology, School of Dentistry, Kyungpook National University, Daegu, South Korea; Texas A&M University Baylor College of Dentistry, UNITED STATES

## Abstract

Osteoblast differentiation can be modulated by variations in order of nanoscale topography. Biopolymers embedded with carbon nanotubes can cause various orders of roughness at the nanoscale and can be used to investigate the dynamics of extracellular matrix interaction with cells. In this study, clear relationship between the response of osteoblasts to integrin receptor activation, their phenotype, and transcription of certain genes on polymer composites embedded with carbon nanotubes was demonstrated. We generated an ultrathin nanocomposite film embedded with carbon nanotubes and observed improved adhesion of pre-osteoblasts, with a subsequent increase in their proliferation. The expression of genes encoding integrin subunits α_5_, α_v_, β_1_, and β_3_ was significantly upregulated at the early of time-point when cells initially attached to the carbon nanotube/polymer composite. The advantage of ultrathin nanocomposite film for pre-osteoblasts was demonstrated by staining for the cytoskeletal protein vinculin and cell nuclei. The expression of essential transcription factors for osteoblastogenesis, such as Runx2 and Sp7 transcription factor 7 (known as osterix), was upregulated after 7 days. Consequently, the expression of genes that determine osteoblast phenotype, such as alkaline phosphatase, type I collagen, and osteocalcin, was accelerated on carbon nanotube embedded polymer matrix after 14 days. In conclusion, the ultrathin nanocomposite film generated various orders of nanoscale topography that triggered processes related to osteoblast bone formation.

## Introduction

The physiochemical properties of biomaterials can influence cell adhesion, cell growth, and subsequent cellular differentiation [1–4]. Therefore, the biochemical, mechanical, and physical properties of an interface can dictate cellular fate. The initial response of transmembrane integrin receptor activation is closely associated with cytoskeleton reorganization and subsequent cellular functions. Genes encoding transcription factors required for osteoblastogenesis and genes that determine the phenotype of osteoblasts are key biomarkers of osteoblast bone-forming processes on biomaterials [5,6].

Surface chemistry modifications modulate the expression of genes encoding integrins, and possibly influence the differentiation of bone cells [7]. The nanoscale roughness and stiffness of biomaterials are two major independent physical factors that can dictate the long-term function of osteoblasts [8–10]. As an example, the order and pattern of topographical cues can affect osteoblast adhesion and proliferation. Specifically, nanoscale island pattern provided greater osteoblast adhesion than those obtained with the nanoscale pit patterns and the microscale island [11]. In another study, the nanotopography of microcavities induced a synergistic effect by different scale stimulus with respect to cell proliferation [12]. A biomimetic hydroxyapatite polymer composite with a favorable nanopatterned surface improved protein adsorption and enhanced compressive modulus [13]. By controlling the island height (13–95 nm) on a polystyrene surface, a relationship between island height and cell responses was examined [14]. It has also been reported that nanotopography can direct mesenchymal stem cells toward the osteoblast lineage through the regulation of microRNA circuits [15]. Although controlling topography at the nanoscale is difficult when the structures on a nanopatterned surface are similar in size to individual cell receptors, the nanotopography of a surface plays a significant role with respect to integrin molecules, as these are critical communication channels through which cells interact with adjacent surfaces [16].

In this regard, polymer composites reinforced with carbon nanotubes (CNTs) can enhance the biocompatibility for various cells types, including bone-forming cells, as the surface roughness can be engineered across a wide range. It has been hypothesized that CNTs can be used to generate a nanoscale surface topography similar to that seen in the bone; this surface has almost identical dimension with linear hydroxyapatite-collagen matrix molecules. Furthermore, CNTs exhibit strong mechanical stiffness, which confers upregulated expression of integrins by mechanical stimulation, and drives the differentiation of stem cells or pre-osteoblastic cells to bone cells [17–19]. In this line, composites of polymers and CNTs can be used to simultaneously generate various nanoscale topographies with various orders of surface stiffness. Especially, polycarbonate urethane (PCU) is denser and harder than ultra-high molecular weight polyethylene (UHMWPE), with a melting temperature of 190–205°C, making it an ideal non-degradable biomaterial for vascular and orthopedic applications. In addition, PCU can provide multiple physiochemical stimuli for intercellular responses, with greater durability than UHMWPE. The properties of PCU, such as roughness and hardness, can be further tailored by the incorporation of CNTs [20,21].

As such, the objective of this study was to elucidate the relationship between nano-topographical gradient using medical grade polymers and associated osteoblast differentiation. To achieve this goal, we demonstrated a strategy for generating CNT/PCU thin-film composites and identified how pre-osteoblasts interact with CNT/PCU structures by examining initial and long term functions of osteoblasts on various nanotopographies.

## Materials and Methods

### Sample preparation

Medical grade PCU was obtained from Lubrizol (PC-3575A), which is FDA-approved and used clinically as a cardiovascular implant biomaterial. PCU does not degrade by oxidation and, thus, highly durable in environments where blood and proteins are present. We used oxidized CNTs (30- to 50-nm diameter; 900–1351, SES, USA) to generate various nanoscale topographies on PCU. PCU was suspended in CHCl_3_ at a concentration of 1 g per 16 mL and was sonicated to enhance the dispersion of PCU. Oxidized CNTs, following acidic, were suspended in CHCl_3_ and sonicated. The PCU and CNT solutions were then mixed at the desired ratio and sonicated. CNT/PCU composites were cast by spin-coating 1 mL of CNT/PCU solution at 3000–5000 rpm. All coated samples were dried under vacuum for 2 days to evaporate CHCl_3_. Samples were stored under sterile conditions, by continuous exposure to UV light, until required.

### Surface characterization

The surface roughness of fabricated samples was determined using a non-contact-mode atomic force microscope (AFM; XE-100, Parks System). Dimensions of the scan field of view were 5 μm × 5 μm (or 10 μm) depending on sample types. Commercially available AFM tips (radius of curvature ≤ 10 nm; PPP-NCHR, Parks System) were used in non-contact mode at a scan rate of 0.5 Hz, a tip height of 125 μm, and a constant force of 42 N/m. Water surface contact angles were measured using a Drop Shape Analysis System (Kruss, Germany) and corresponding DSA1 software (Kruss) under ambient conditions. The contact solvent was 3 μL of distilled water. All data were obtained 5 sec after placing the droplet on the respective surfaces.

### Protein quantitation and vitronectin assays

Serum proteins (FBS, Gibco) were diluted (1:5) in phosphate-buffered saline (PBS), adsorbed to flat PCU and CNT/PCU composites of varying ratios, and stored for 4 hrs in a cell culture incubator to emulate the cell adhesion environment. Then, protein solutions were removed using a solution of 2% (w/v) sodium dodecyl sulfate (SDS; L3771, Sigma). The concentration of proteins was determined with a Coomassie Plus (Bradford) Assay Kit (23236, Sigma). The absorbance at 595 nm was measured using an Asys UVM 340 spectrophotometer (Biochrom). Protein concentrations were determined by extrapolation from a standard curve for albumin, and were normalized to glass.

Human vitronectin (VN; V8379, Sigma) adsorbed on PCU, 10% CNT/PCU, and 50% CNT/PCU surfaces under standard cell culture conditions were used to evaluate VN adsorption. After 4 hrs, VN was removed with 2% (w/v) SDS (L3771, Sigma) and surfaces gently washed three times. The adsorbed VN on sample surfaces was measured with a Coomassie Plus (Bradford) Assay Kit (23236, Thermo) at 595 nm using an Asys UVM 340 spectrophotometer. The amount of VN in solution was calculated by extrapolation from a standard curve generated for VN.

### Osteoblast cell culture

We used a murine calvaria-derived pre-osteoblast cell line, MC3T3-E1 subclone 4, from the American Type Cell Culture Collection (ATCC, Manassas, VA, USA) in this study. Cells were cultured in α-minimum essential medium (Gibco BRL Life Technologies, Grand Island, NY, USA) supplemented with 10% fetal bovine serum (FBS; Gibco BRL Life Technologies), 100 units/mL penicillin (Keunhwa Pharmaceutical, Seoul, Korea), and 100 units/mL streptomycin (Donga Pharmaceutical, Seoul, Korea). Cultures were incubated at 37°C/5% CO_2_ with 100% humidity. Culture media were changed every other day and cultures passaged using 0.05% trypsin/0.02% EDTA before they were confluent. Cell culture experiments were independently repeated three times.

### Attachment and viability assays

To evaluate cell attachment and viability, cells were cultured on various surfaces at an initial seeding density of 4 × 10^4^ cells/well. Cell attachment and viability were evaluated after 4 hrs and 2 days in culture. Cell adhesion and viability were determined using 3-(4,5-dimethylthiazol-2-yl)-2,5-diphenyltetrazolium bromide assays (MTT; M-2128, Sigma). MTT (10 mg/mL) was added into each well containing cells and allowed to incubate for 4 hrs. Isopropanol in 0.04 M HCl was added to dissolve formazan crystals and the absorbance at 570 nm was determined using an Asys UVM 340 spectrophotometer (Biochrom). Cell adhesion and viability were normalized as the relative absorbance for each sample compared to controls.

### Evaluation of osteoblast morphology

Osteoblast morphology, cytoskeletal arrangement, and focal adhesion of cells were observed using disk-based confocal scanning microscopy (DCSM; BX51, Olympus, Japan) and Meta-morph software (Olympus, Japan). Cells samples were examined after culturing for 4 and 24 hrs. Osteoblasts were cultured on various surfaces at an initial seeding density of 5000 cells/cm^2^. To investigate the effects of nanotopographical features on focal adhesion and spread of osteoblasts, distribution of vinculin, and organization of actin filaments were investigated by DCSM. Focal adhesion contacts and cytoskeletons were identified by incubating samples with a monoclonal antibody against vinculin (Sigma-Aldrich, St. Louis, MO, USA) and fluorescein isothiocyanate (FITC)-labeled phalloidin (Sigma-Aldrich). An appropriate secondary goat-anti-mouse antibody was used to visualize vinculin staining (Invitrogen, Carlsbad, CA, USA). Samples were mounted on glass slides using anti-fade reagent and 4,6-diamidino-2-phenylindole dihydrochloride (DAPI; Invitrogen), and examined by DCSM.

### Quantitative polymerase chain reaction (qPCR) assays

To evaluate the mRNA levels of various integrins (α_1_, α_2_, α_ν_, α_5_, _1,_ and _3_), cells were seeded on 15 different surfaces in 6-well culture plates at an initial seeding density of 4 × 10^4^ cells/well and cultured for 4 and 24 hrs. To evaluate the mRNA levels of Runt-related transcription factor 2 (*Runx2*), Sp7 transcription factor 7 (also known as osterix, *Sp7*), type I collagen (*Col1*), alkaline phosphatase (*Alpl*), integrin binding sialoprotein (also known as bone sialoprotein, *Ibsp*), secreted phosphoprotein 1 (also known as osteopontin, *Spp1*), bone gamma-carboxyglutamic acid protein (also known as osteocalcin, *Bglap*), and glyceraldehyde-3-phosphate dehydrogenase (*Gapdh*), cells were seeded on seven different surfaces in 6-well culture plates at an initial seeding density of 4 × 10^4^ cells/well and cultured for 7 and 14 days. Total RNA was extracted using Trizol reagent (Gibco BRL Life Technologies) after the appropriate incubation time for cultures, and RNA samples were quantified. To synthesize first-strand complementary DNA, reverse transcription was performed as described previously [22]. We then conducted qPCR assays as described previously [22] using the oligonucleotide primers shown in Table 1. The reference gene *Gapdh* was used as an internal control gene. Data were analyzed using the 2^−ΔΔCT^ method and normalized against *Gapdh* expression levels. The results of the expression of various genes are shown as fold differences of the gene expression relative to the results of the PCU surface.

**Table 1:** Oligonucleotide primers used in qPCR assays.

Target	Primer sequences
Mouse gene symbol	
*Itga1*	Forward primer 5′–3′: ACA CTC GGT GAC CTT GTG GAT
	Reverse primer 5′–3′: ACA ATT CCA GCA ACC ACG CCT
*Itga2*	Forward primer 5′–3′: GGA CTG CAG AAC CAC TTC CT
	Reverse primer 5′–3′: AGC GGC AGA GAT CGA TAC AC
*Itga5*	Forward primer 5′–3′: GGC AGA AGG CAG CAA TGG TG
	Reverse primer 5′–3′: AGG CAT CTG AGG TGG CTG GA
*Itgav*	Forward primer 5′–3′: CAT CTT GGC AGT TCT CGC AG
	Reverse primer 5′–3′: GCG CCA CTT AAG AAG CAC CT
*Itgb1*	Forward primer 5′–3′: TTA TTG GCC TTG CCT TGC TG
	Reverse primer 5′–3′: CCG CCT GAG TAG GAT TCA TT
*Itgb1*	Forward primer 5′–3′: ATG AAT GCG CAG CAC AGA GC
	Reverse primer 5′–3′: CAG GAA GGC GCG TAA GCA AT
*Runx2*	Forward primer 5′–3′: TAA GAA GAG CCA GGC AGG TG
	Reverse primer 5′–3′: TGG CAG GTA CGT GTG GTA GT
*Sp7*	Forward primer 5′–3′: TCA CTT GCC TGC TCT GTT CC
	Reverse primer 5′–3′: GCG GCT GAT TGG CTT CTT CT
*Col1*	Forward primer 5′–3′: ATC CAA CGA GAT CGA GCT CA
	Reverse primer 5′–3′: GGC CAA TGT CTA GTC CGA AT
*Alpl*	Forward primer 5′–3′: CTT GAC TGT GGT TAC TGC TG
	Reverse primer 5′–3′: GAG CGT AAT CTA CCA TGG AG
*Spp1*	Forward primer 5′–3′: TCA AGT CAG CTG GAT GAA CC
	Reverse primer 5′–3′: CTT GTC CTT GTG GCT GTG AA
*Ibsp*	Forward primer 5′–3′: GGA GGA GAC AAC GGA GAA GA
	Reverse primer 5′–3′: CCA TAC TCA ACG GTG CTG CT
*Bglap*	Forward primer 5′–3′: TGC TTG TGA CGA GGT ATC AG
	Reverse primer 5′–3′: GTG ACA TCC ATA CTT GCA GG
*Gapdh*	Forward primer 5′–3′: GGC ATT GCT CTC AAT GAC AA
	Reverse primer 5′–3′: TGT GAG GGA GAT GCT CAG TG

*Itg*: integrin, *Sp7*: Sp7 transcription factor 7, *Col1*: type I collagen, *Alpl*: alkaline phosphatase, liver/bone/kidney, *Spp1*: secreted phosphoprotein 1, *Ibsp*: integrin binding sialoprotein, *Bglap*: bone gamma-carboxyglutamic acid protein, *Gapdh*: glyceraldehyde-3-phosphate dehydrogenase

### Statistical analysis

Statistical differences among samples were determined by analysis of variance (ANOVA) followed by the Student–Newman–Keuls multiple comparison test. Student’s *t*-test was used to compare two groups. *, **, and *** indicated that significance of p values were less than 0.05, 0.01, and 0.001, respectively. At least three samples were tested from three independent experiments. All results are presented as the mean ± SEM with *n = 3*.

## Results

### Generating topography on polymer thin films using CNTs

**Fig 1:**
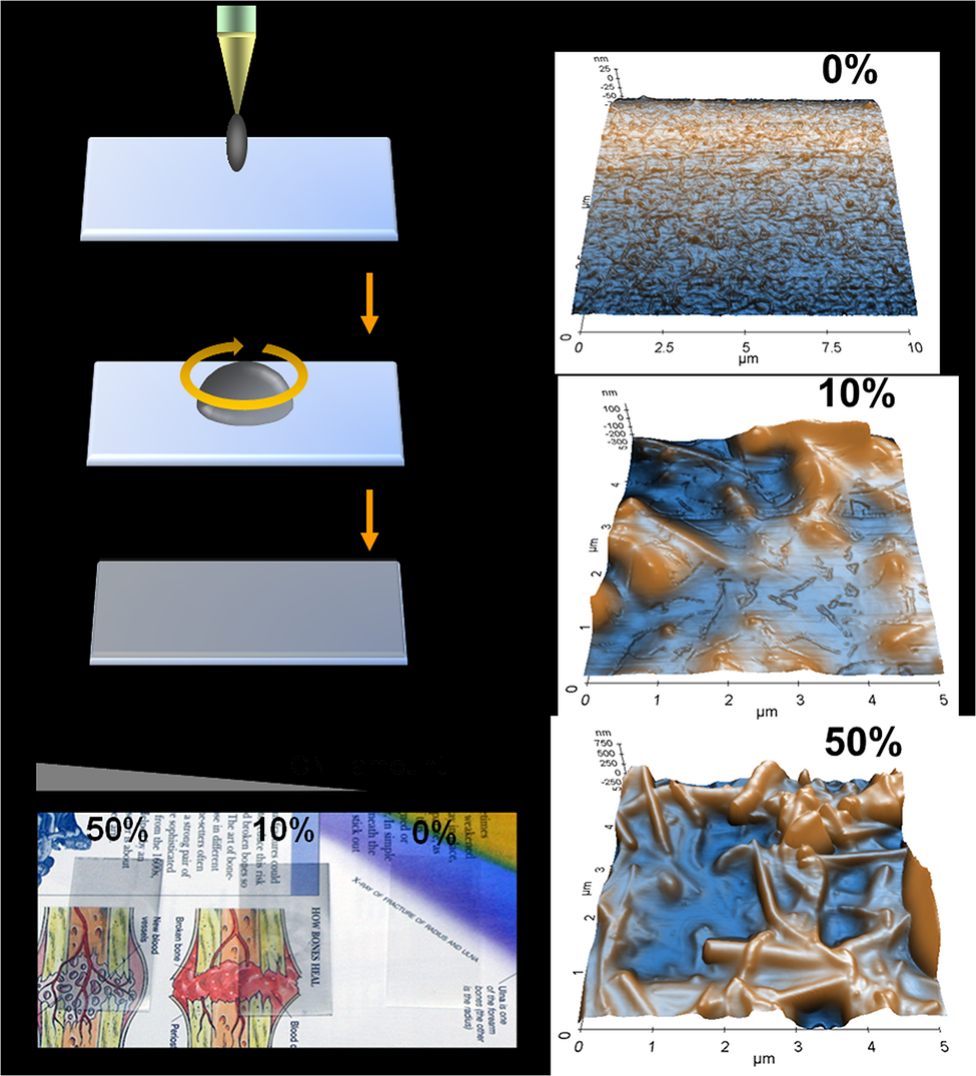
Fabrication, surface transparency, and surface morphology of CNT/PCU composite thin film. (a) A schematic showing the fabrication of a CNT/PCU composite thin film using spin casting techniques. (b) Transparency of PCU, 10% CNT/PCU, and 50% CNT/PCU. The CNT/PCU composites were made transparent under visible and optical microscopy. (c). Nanoscale surface topography of PCU, 10% CNT/PCU, and 50% CNT/PCU, as determined by AFM. An increase in the presence of nanostructures corresponded with increasing levels of CNTs embedded in PCU.

**Fig 2:**
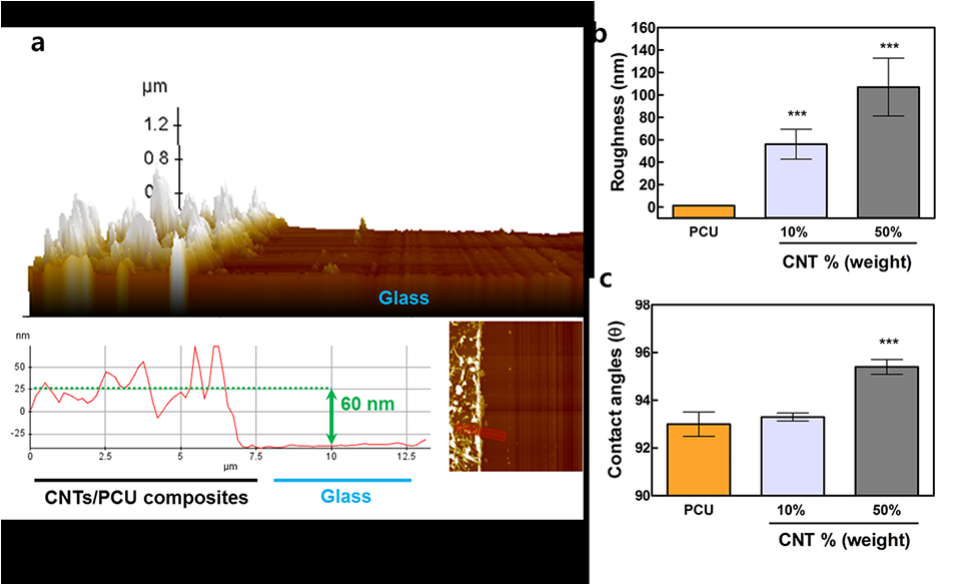
Thickness, roughness, and contact angle of CNT/PCU composites. (a) The film thickness was 60 nm for CNT/PCU. The thickness of the pure PCU surface was around 100 nm (data not shown). (b) Roughness (RMS) analysis of PCU (orange) and CNT/PCU composites (gray for 10% CNT and dark gray for 50% of CNT in PCU) showed increased nanoscale roughness as the quantity of CNTs embedded in PCU increased. (d) Goniometry revealed increase in contact angle with increase of CNT amount in PCU matrix. All data represent the mean ± SEM (*n* = 3). *p < 0.05, **p < 0.01, and ***p < 0.001 *vs*. control (PCU).

Using the suggested fabrication method (Fig 1A), two different orders of nanoscale surface topography were generated under optically transparent conditions (Fig 1A and 1B). Total thickness was less than 100 nm for all samples (Fig 2A). Surface roughness was increased by embedding CNTs mixed with medical grade PCU as identified by AFM (Figs 1C and 2C). Increasing quantities of CNTs in PCU resulted in a change of the surface contact angle (Fig 2C). The increment in contact angle (hydrophobicity) might be due to exposure of hydrophobic CNTs, as CNT exposure on glass also increased contact angles (data not shown). Nanoscale surface roughness ranged from 40 to 120 nm, with increasing quantities of CNTs on PCU corresponding with increased surface roughness (Figs 1C and 2B). The thickness of our transparent CNT/PCU composite was about 60 nm (Fig 2A).

### Pre-osteoblast adhesion levels, morphological and cytoskeleton analyses, and expression levels of integrin genes after 4 hrs

**Fig 3:**
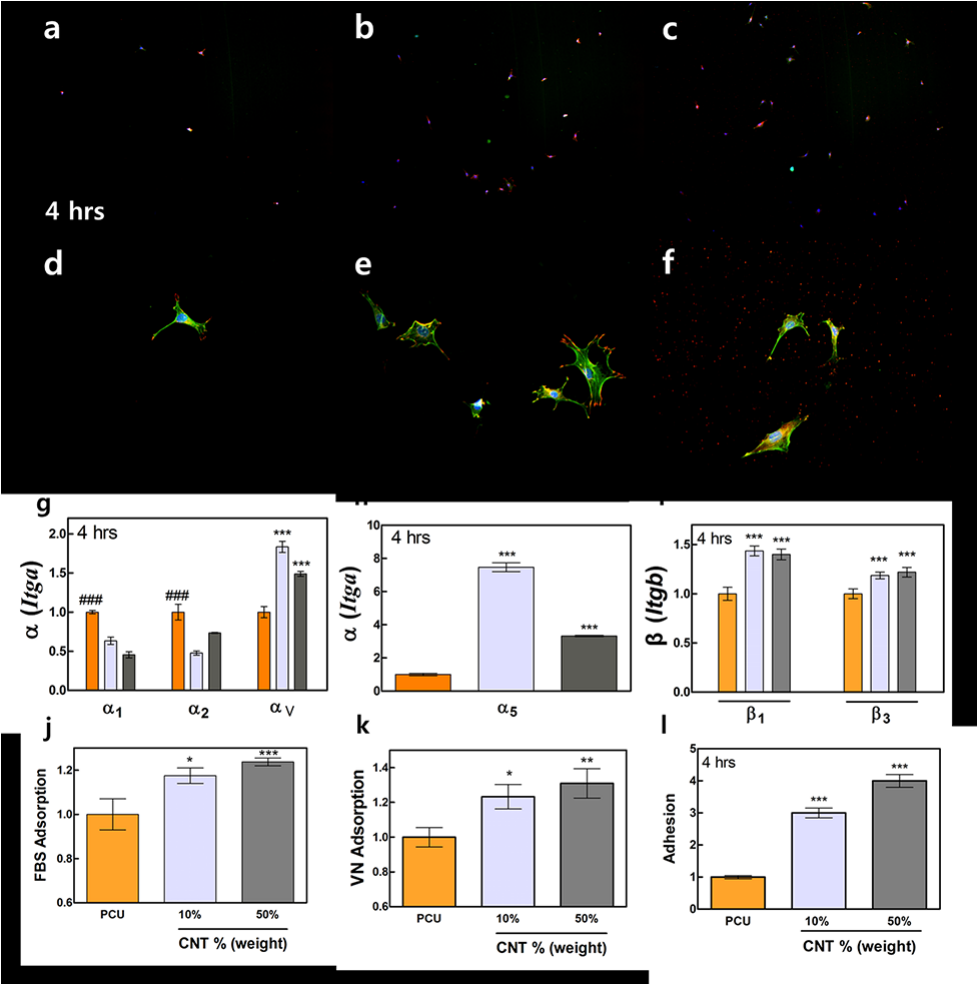
Pre-osteoblast adhesion, cytoskeletal organization, and focal adhesion on PCU and CNT/PCU composites. (a) The actin cytoskeleton (green) and focal adhesions (red) of pre-osteoblasts grown on the PCU (a, d), 10% CNT/PCU (b, e), and 50% CNT/PCU (c, f) surfaces after incubation for 4 hrs. (g-i) Relative mRNA expression levels of fold change of the integrin subunits α_1,_ α_2_, α_5_, α_v_, β_1,_ and β_3_. Pre-osteoblasts were grown on the pure PCU (orange) surface and the two CNT/PCU composite surfaces (gray for 10% CNT and dark gray for 50% of CNT in PCU). mRNA expression levels were determined using qPCR assays after 4-h culture. (j-k) Fold change of FBS and VN adsorption. (l) Fold change of pre-osteoblast cell adhesion levels on the CNT/PCU surfaces compared with that on the PCU surface after 4 hrs. All data represent the mean ± SEM (*n* = 3). *p < 0.05, **p < 0.01, ***p < 0.001 *vs*. control (PCU) and ^###^ *p* < 0.001 *vs*. CNT/PCU composites.

Adhesion of pre-osteoblasts and their spread on various surfaces were determined by confocal microscopy (Fig 3A–3F and 3L). The 10% and 50% CNT/PCU surfaces exhibited significantly greater levels of cell attachment than the pure PCU surface (Fig 3A–3C and 3L). Cell attachment levels for the 50% CNT/PCU surface were 25% higher than those for the 10% CNT/PCU composite and 400% higher than those for pure PCU (Fig 3L). These findings suggest that increase in roughness of the surface due to increase in CNT content provides conditions that promoted adhesion of anchorage-dependent osteoblastic cells. Expression levels of the mRNA transcripts for the integrin α_v_ and α_5_ subunits significantly increased for both CNT/PCU composites (Fig 3G–3H). We observed upregulated expression levels of the α_1_ and α_2_ subunits for the pure PCU surface. Especially, expression levels of integrin α_5_ on the 10% CNT/PCU composite were notably increased after 4 hrs (Fig 3H). Expression of the β integrin subunits, β_1_ and β_3_, was upregulated for both the 10% and 50% CNT/PCU surfaces (Fig 3I). According to the previous studies, role of integrin genes on the subsequent osteoblast differentiation [23–28] was discussed. In this study, up-regulation of specific integrin genes on CNT/PCU surfaces (at 4 hrs) may trigger later osteoblast differentiation through activating integrin-mediated signaling pathways. Preliminary time dependent mRNA expression of integrins confirmed that integrins reached the highest value at 4 hrs (S2 Fig)

The extent of total FBS and human VN adsorption increased as the ratio of CNTs to PCU increased (Fig 3J and 3K). It is possible that increased surface area and surface topography were simultaneously involved in greater degrees of protein adsorption. A greater number of VN-binding sites were observed on the 10% CNT-PCU surfaces, corresponding with increased α_5_ integrin expression levels (Fig 3H and S1 Fig). Total FBS and VN adsorption, expression of β_1_, β_3_ subunits, and extent of cell adhesion all increased on the CNT-PCU composites (Fig 3I–3L).

### Pre-osteoblast proliferation levels, morphological and cytoskeleton analyses, and integrin gene expression levels after 24 hrs

**Fig 4:**
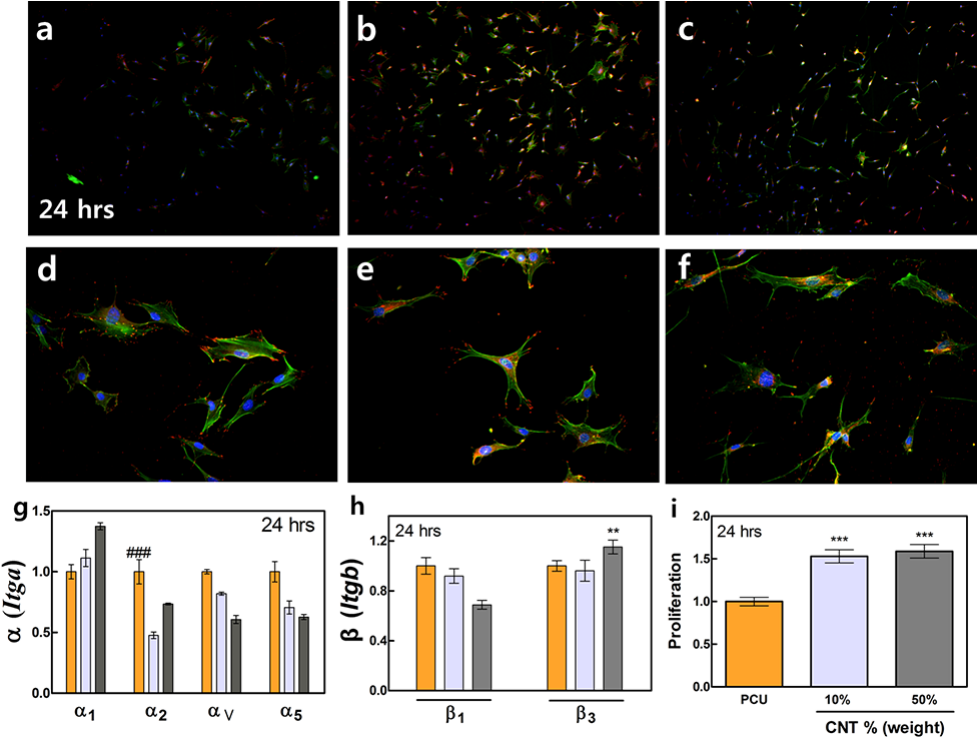
Pre-osteoblast proliferation, integrin activation, cytoskeletal organization, and focal adhesion on PCU and CNT/PCU composites. (a) Actin cytoskeleton (green) and focal adhesions (red) of pre-osteoblasts grown on the PCU (a, d), 10% CNT/PCU (b, e), and 50% CNT/PCU (c, f) surfaces after 24 hrs. (g) Fold change of mRNA expression levels of α_1,_ α_2_, α_5,_ and α_v_ integrin and of (h) β_1_ and β_3_ integrin in pre-osteoblasts grown on the PCU (orange), 10% CNT/PCU (gray), and 50% CNT/PCU (dark gray) surfaces were assessed by qPCR after 24 hrs in culture. (i) Fold change of pre-osteoblast cell proliferation on CNT/PCU composites compared with that on the pure PCU surface after 24 hrs. All data represent the mean ± SEM (*n* = 3). *p < 0.05, **p < 0.01, ***p < 0.001 *vs*. control (PCU) and ^###^ *p* < 0.001 *vs*. CNT/PCU composites.

Cytoskeleton analysis showed that pre-osteoblasts were fully attached to the surfaces and the early stages of proliferation was confirmed (Fig 4D–4F). A greater number of focal adhesion contacts (vinculin) and well-defined, elongated cytoskeletons (f-actin) were observed in pre-osteoblasts grown on the 10% and 50% CNT/PCU composites than on the pure PCU surface (Fig 4G–4I). Increase in the level of cell adhesion on CNT-modified surfaces was followed by an increase in the number of proliferating cells (Fig 4I). Cell proliferation on the 10% and 50% CNT/PCU surfaces was greater than on the control PCU surface, with no difference observed between the two CNT/PCU surfaces (Fig 4I). Generation of the cell cytoskeleton was more evident in cells on the 10% CNT/PCU surface. Expression of the α_1_ integrin subunit was upregulated on the 50% PCU/CNT surfaces at 24 hrs (Fig 4G); however, α_v_ and α_5_ expression levels decreased at 24 hrs (Fig 4G). Expression of the β_3_ integrin subunit remained upregulated on the 50% CNT/PCU composites, while β_1_ expression decreased (Fig 4G). The number of vinculin sites at 24 hrs was higher than that at 4 hrs on all surfaces due to increased cell proliferation. The 10% CNT/PCU composite contained the greatest number of vinculin sites (see S1 Fig).

### Osteoblast gene expression

**Fig 5:**
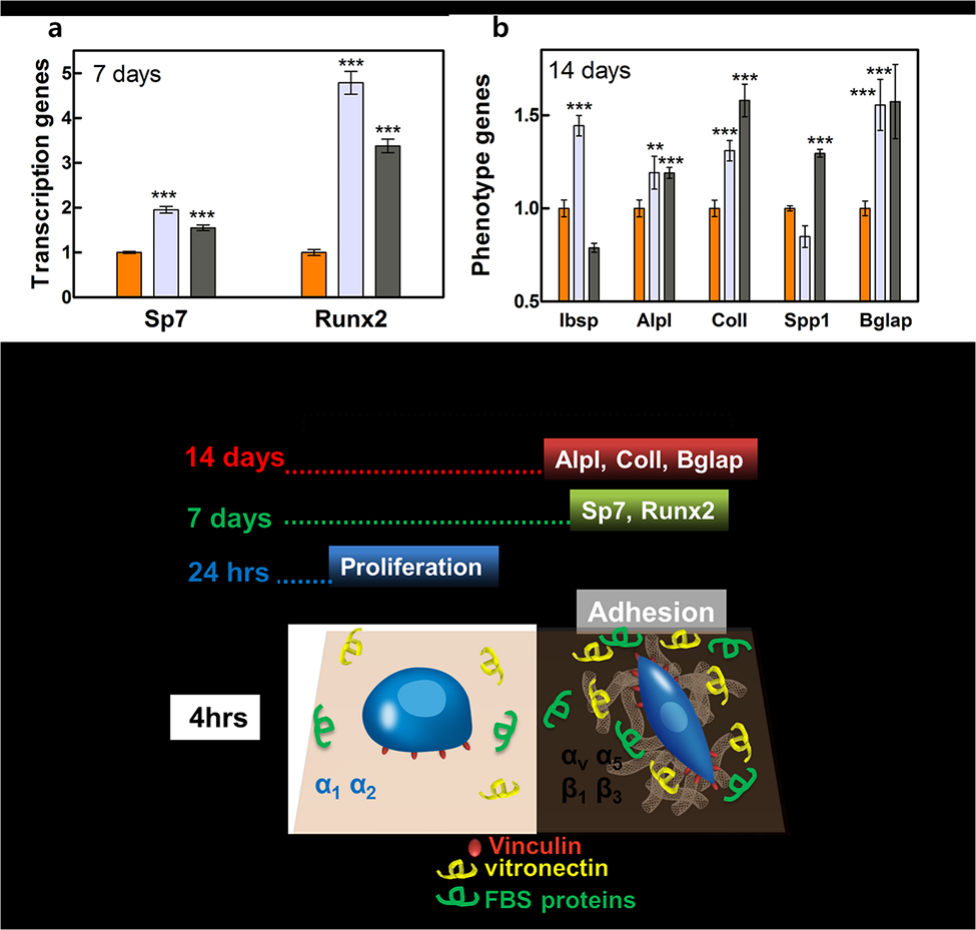
Transcriptional and phenotype gene expression of osteoblasts on PCU and CNT/PCU composites. The mRNA levels of (a) *Sp7* and *Runx2* at 7 days and (b) *Ibsp*, *Alpl*, *Col1*, *Spp1*, and *Bglap* after 14 days in osteoblasts grown on the PCU (orange) and CNT/PCU composite surfaces (gray for 10% CNT and dark gray for 50% of CNT in PCU) were determined by qPCR. (c) Dominant biomarkers of osteoblast responses (short and long term). All data represent the mean ± SEM (*n* = 3). *p < 0.05, **p < 0.01, ***p < 0.001 *vs*. control (PCU).

Expression levels of *Runx2* and *Sp7* after 7 days and of *Col1*, *Ibsp*, *Alpl*, *Spp1*, and *Bglap* after 14 days increased in cells grown on the CNT/PCU composites (Fig 5A and 5B). Expression of *Runx2* and *Sp7* mRNAs at 7 days was highest on the 10% CNT/PCU surface (Fig 5A). Increased *Sp7* and *Runx2* expression on the CNT/PCU composites resulted in a corresponding increase in *Col1*, *Ibsp*, *Alpl*, *Spp1*, and *Bglap* expression (Fig 5B). Osteoblasts on the CNT/PCU surfaces contained greater levels of *Col1* mRNAs than those on the pure PCU surface at 14 days (Fig 5B). Increased *Col1* expression on the CNT/PCU surfaces indicated that pre-osteoblasts had entered a more mature stage than cells on the PCU surface and had commenced differentiation toward osteoblasts (Fig 5B). Expression levels of *Bglap*, a terminal marker of osteoblast differentiation, were significantly higher in cells grown on the CNT/PCU surfaces than in those on the PCU surfaces at 14 days (Fig 5B), indicating a more fully differentiated stage of cells on the CNT/PCU samples. Expression of *Ibsp*, *Alpl*, *and Spp1*, early markers of osteoblast differentiation, was also upregulated in cells on the CNT/PCU composites at 14 days (Fig 5B). Taken together, our findings indicate that the nanoscale topography of CNT composites promotes osteoblast differentiation to a greater extent than that of the PCU surface (Fig 5C). The expression levels of key osteoblastogenesis markers those are dominant at each time point have been summarized (Fig 5C).

## Discussion and Summary

Surface chemistry, stiffness, and nanoscale topography are all significant factors for directing integrin-mediated cell interactions [29]. However, the effects of integrins associated with different nanoscale surface features on osteoblast differentiation for CNT-embedded polymers remains unclear. It was previously shown that a monolayer of CNTs was sufficient for triggering the adhesion of osteoblasts [30]. At present, it is difficult to measure the responses of osteoblasts to nanoscale medical grade polymers. As such, we attempted to determine the effects of biocompatible materials on integrin expression and differentiation of osteoblasts. The α_v_ and α_5_ subunits of integrin play a critical role in the early stages of cellular adhesion and subsequent differentiation of pre-osteoblasts on CNT/PCU composite surfaces, while previous titanium surface promoted proliferation and differentiation of osteoprogenitor cells by increased expression of integrins α_2_ and α_5_ [31,32].

The initial cell attachment to the 50% CNT/PCU surface was significantly greater than that for PCU surfaces. The rougher surface, corresponding to the greater proportion of CNTs on the 50% CNT/PCU surface, allowed for a greater degree of initial cell attachment. Similar results were seen with respect to FBS and VN adsorption; the highest degree of adsorption after 4 hrs was observed for the 50% CNT/PCU composite. However, proliferation rate (viability after 24 hrs/ initial adhesion at 4 hrs) of PCU was greater than 10% and 50% of CNT/PCU composite (Fig 5C). This might interpreted that CNT/PCU composite already entered differentiation before 24 hrs. In contrast, PCU still do not sufficiently entered differentiation stage and keeping pre-osteoblastic proliferation stage (Fig 5).

Analysis of *Sp7* and *Runx2* expression levels using qPCR assays were consistent with the rate of initial cell adhesion. Expression of *Sp7* and *Runx2* in cells grown on both CNT/PCU surfaces increased over time. For cells grown on the PCU surface, expression levels of these transcription factors relatively lower than CNT/PCU surfaces. Expression of *Alpl*, *Col1*, *and Bglap* was significantly upregulated on both CNT/PCU surfaces after 14 days, while *Ibsp* and *Spp1* were selectively upregulated after 14 days. The *Ibsp* expression levels were higher on the 10% CNT/PCU surface than the 50% CNT/PCU composite at 14 days. Although BSP and osteopontin were known as osteoblast differentiation makers, their action mode to bone formation is not clearly elucidated yet. Specifically, both positive and negative aspects of osteopontin were discussed during the early stage of osteoblast differentiation and bone formation process [33–38]. Furthermore, inconsistent trend of osteocalsin and osteopontin gene expression (MC3T3-E1 cells) was observed on modified titanium surfaces [39]. In conclusion, osteocalcin is a critical terminal maker for osteoblast differentiation, while osteopontin can contribute up-regulation of osteoblast genes at the early stage. Considering all expression levels of other osteoblast differentiation markers, it was concluded that a certain aspect of the nanotopography of surfaces embedded with CNTs induced faster bone formation.

Although generating nanotopographies using CNTs modulated surface roughness, further investigation is required to analyze more wide sets of surface roughness and associate osteoblast response. The reason is that too strong adhesion by increased nanoscale roughness may hinder cell proliferation [9,40–42].

Controlling surface topography at the nanoscale could be a novel technique for observing the growth, movement, and function of cells. Specifically, obtained results could be used to accelerate bone formation at the barrier membrane during guided bone regeneration (GBR) surgery. In addition, transparent surfaces are useful for further investigating live cell dynamics on the surfaces of biomaterials. Quantitative and qualitative investigation using live cell microscopy can provide valuable insights into cell fate, changes in morphology, and cellular proliferation. Future studies will employing the real-time analysis of bone cell responses to transparent polymer nanotopography should provide more evidence of these features.

In this study, we controlled various features of polymer surface nanotopography by using CNTs. We concluded that osteoblast differentiation was initiated when the expression of the integrins α_v_, α_5,_ β_1,_ and β_3_ was induced by binding to CNT/PCU composites. The expression levels of genes associated with osteoblast phenotype were consistently higher on the two CNT/PCU composites we generated than on the pure PCU surface.

## Supporting Information

S1 Fig Areas of vinculin sites on PCU and CNT/PCU composites at 4 and 24 hrs. (TIF)

S2 Fig Time dependent alpha and beta integrin activation reaches the maximum at 4 hrs and decreasing after 4 hrs. (TIF)
