# Correction: Analysis of Osteoblast Differentiation on Polymer Thin Films Embedded with Carbon Nanotubes

**DOI:** 10.1371/journal.pone.0132874

**Published:** 2015-07-13

**Authors:** Jin Woo Lee, Jin-Woo Park, Dongwoo Khang

Portions of Figs 1, 3 and 5 are illegible due to their black backgrounds. Please see the corrected figures here.

**Fig 1:**
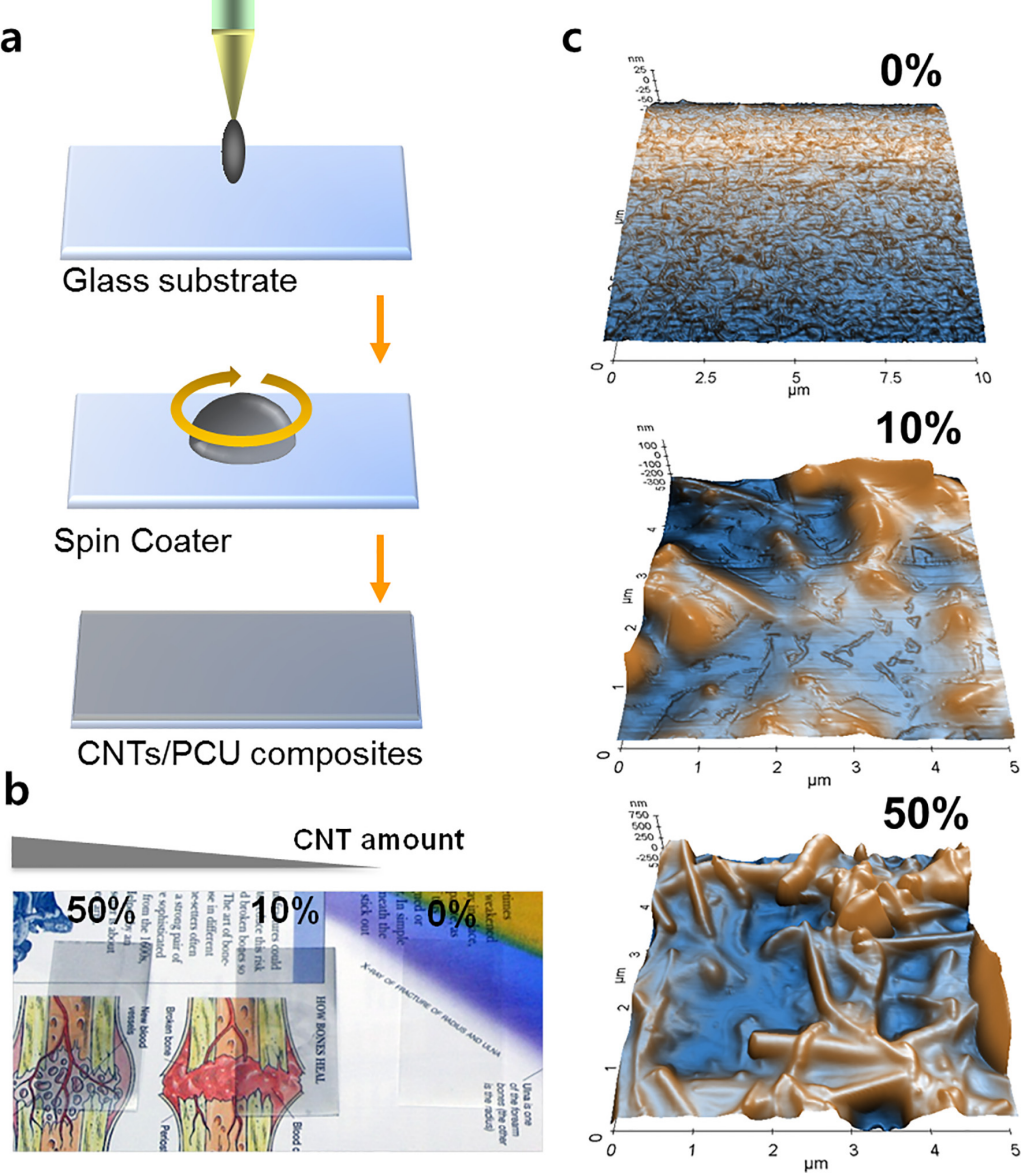
Fabrication, surface transparency, and surface morphology of CNT/PCU composite thin film. (a) A schematic showing the fabrication of a CNT/PCU composite thin film using spin casting techniques. (b) Transparency of PCU, 10% CNT/PCU, and 50% CNT/PCU. The CNT/PCU composites were made transparent under visible and optical microscopy. (c). Nanoscale surface topography of PCU, 10% CNT/PCU, and 50% CNT/PCU, as determined by AFM. An increase in the presence of nanostructures corresponded with increasing levels of CNTs embedded in PCU.

**Fig 3:**
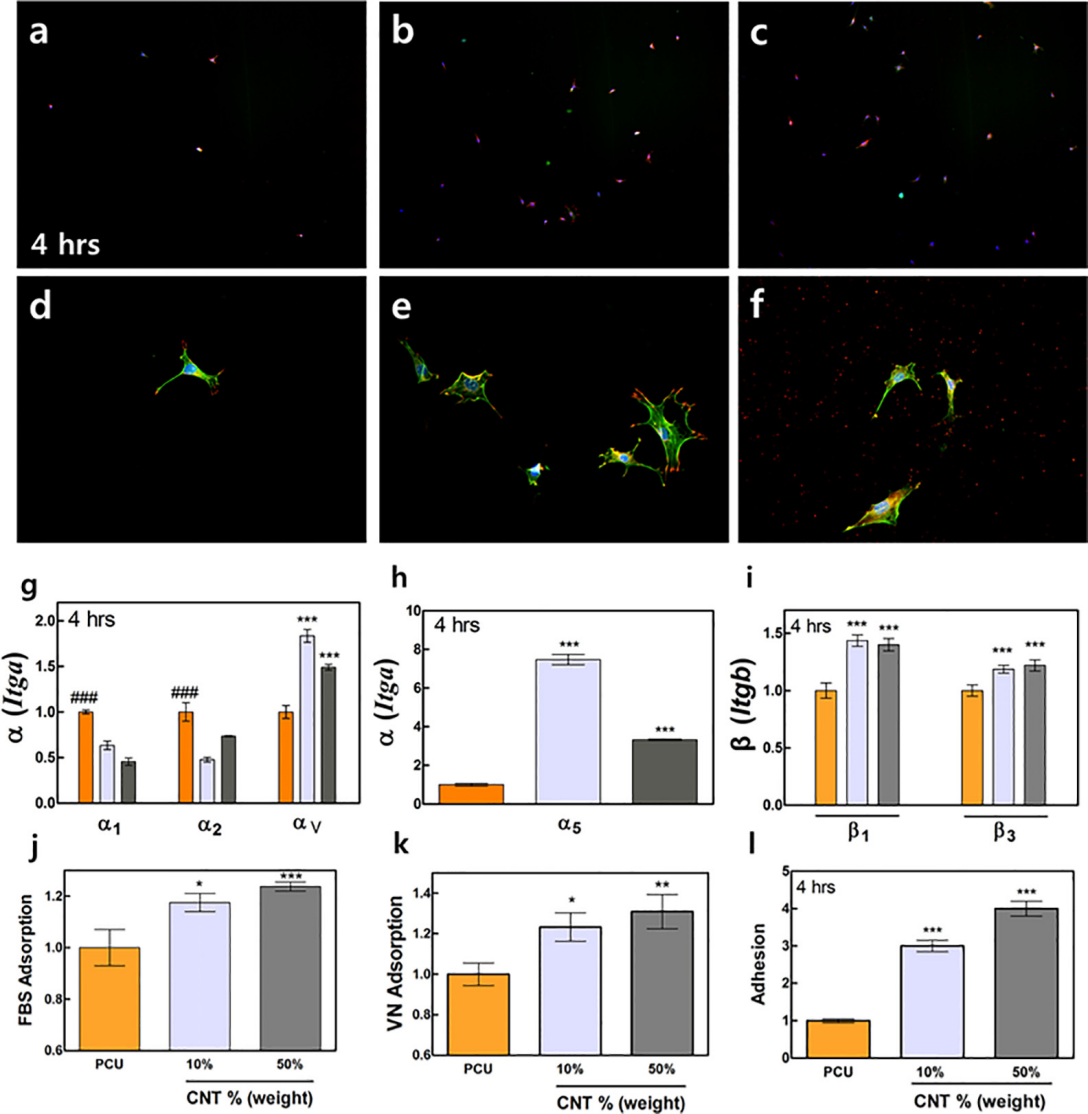
Pre-osteoblast adhesion, cytoskeletal organization, and focal adhesion on PCU and CNT/PCU composites. (a) The actin cytoskeleton (green) and focal adhesions (red) of pre-osteoblasts grown on the PCU (a, d), 10% CNT/PCU (b, e), and 50% CNT/PCU (c, f) surfaces after incubation for 4 hrs. (g-i) Relative mRNA expression levels of fold change of the integrin subunits α_1,_ α_2_, α_5_, α_v_, β_1,_ and β_3_. Pre-osteoblasts were grown on the pure PCU (orange) surface and the two CNT/PCU composite surfaces (gray for 10% CNT and dark gray for 50% of CNT in PCU). mRNA expression levels were determined using qPCR assays after 4-h culture. (j-k) Fold change of FBS and VN adsorption. (l) Fold change of pre-osteoblast cell adhesion levels on the CNT/PCU surfaces compared with that on the PCU surface after 4 hrs. All data represent the mean ± SEM (*n* = 3). ^*^ *p* < 0.05, ^**^ *p* < 0.01, ^***^ *p* < 0.001 *vs*. control (PCU) and ^###^ *p* < 0.001 *vs*. CNT/PCU composites.

**Fig 5:**
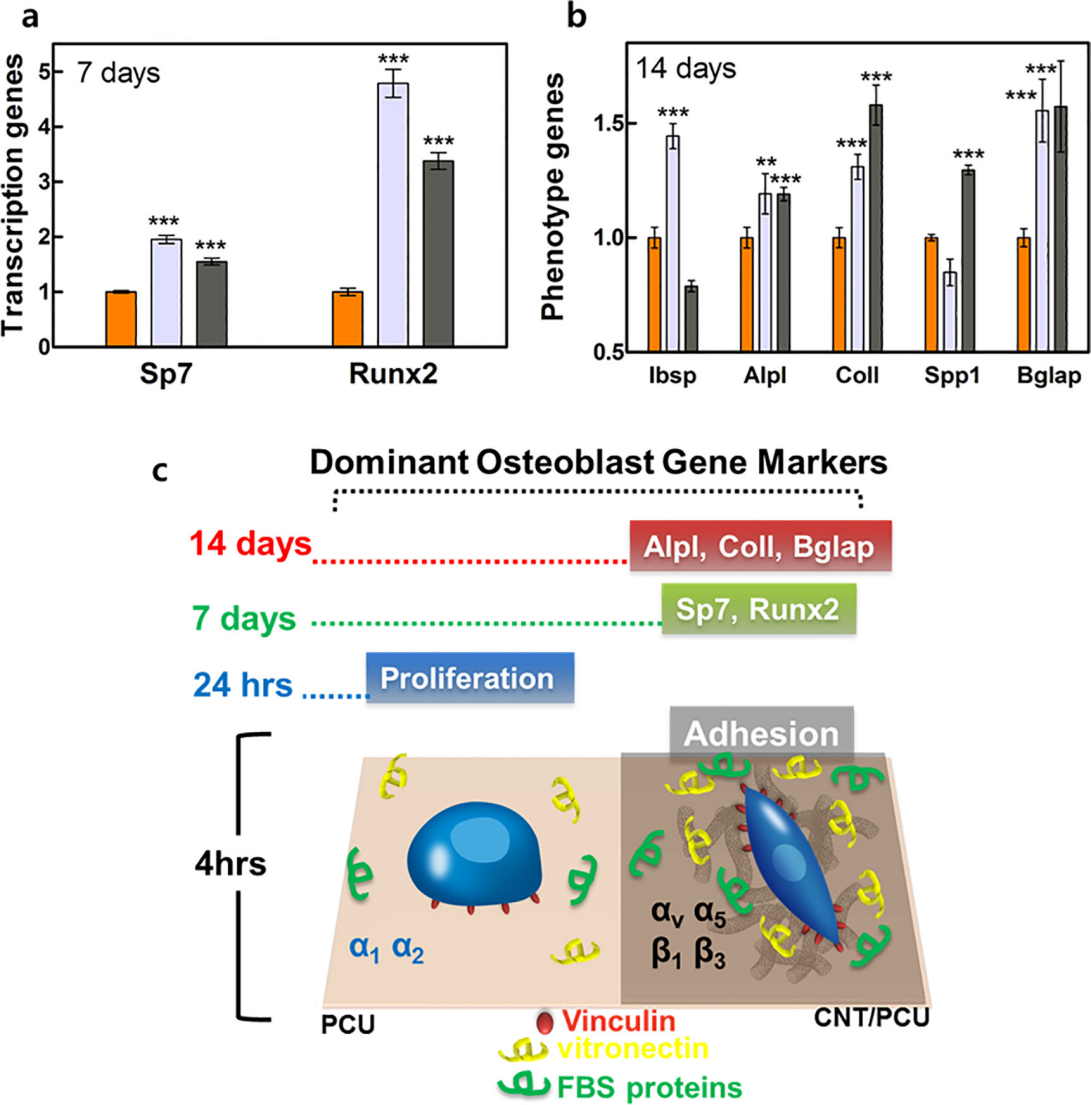
Transcriptional and phenotype gene expression of osteoblasts on PCU and CNT/PCU composites. The mRNA levels of (a) *Sp7* and *Runx2* at 7 days and (b) *Ibsp*, *Alpl*, *Col1*, *Spp1*, and *Bglap* after 14 days in osteoblasts grown on the PCU (orange) and CNT/PCU composite surfaces (gray for 10% CNT and dark gray for 50% of CNT in PCU) were determined by qPCR. (c) Dominant biomarkers of osteoblast responses (short and long term). All data represent the mean ± SEM (*n* = 3). ^*^ *p* < 0.05, ^**^ *p* < 0.01, ^***^ *p* < 0.001 *vs*. control (PCU).
