# Supplementary figures and images for: Analysis of Osteoblast Differentiation on Polymer Thin Films Embedded with Carbon Nanotubes
Source: PLoS One. 2015 Jun 15;10(6):e0129856. doi: 10.1371/journal.pone.0129856 (PMC4468207; doi:10.1371/journal.pone.0129856)

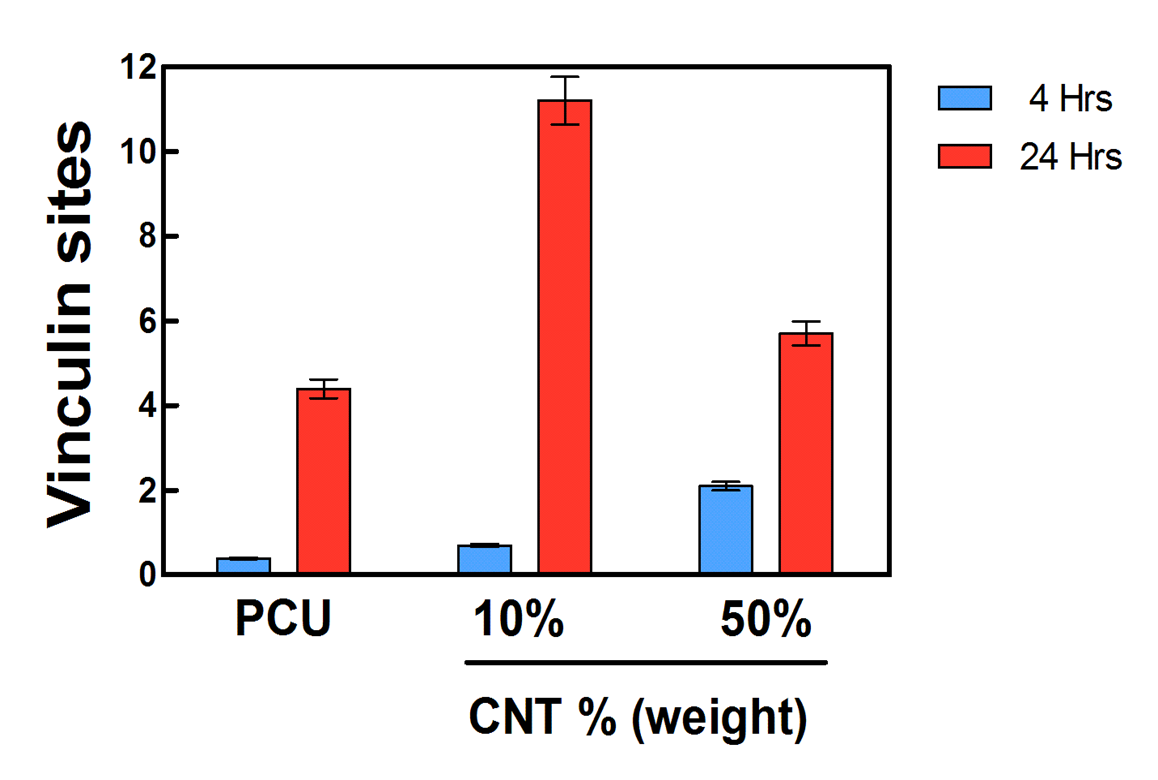

Supplement: S1 Fig — (TIF) [file pone.0129856.s001.tif]

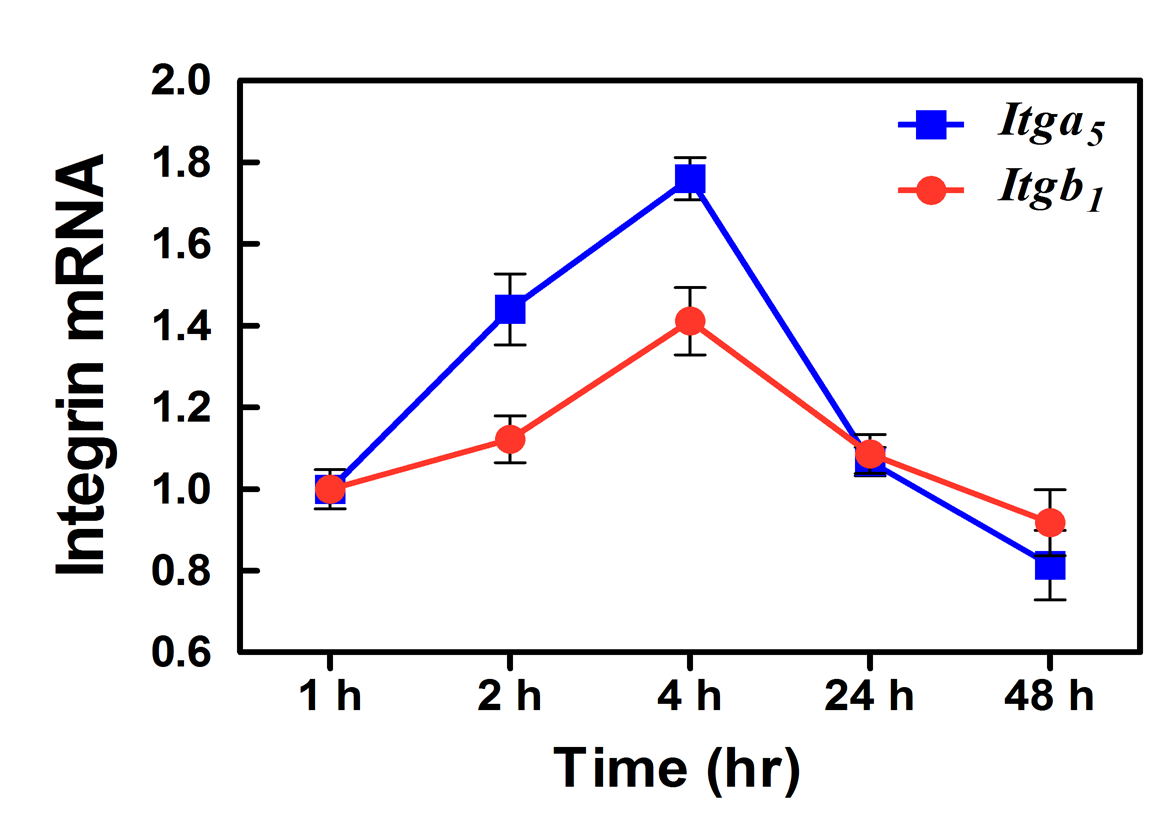

Supplement: S2 Fig — (TIF) [file pone.0129856.s002.tif]
